# Cognitive Dysfunctions in Glaucoma: An Overview of Morpho-Functional Mechanisms and the Impact on Higher-Order Visual Function

**DOI:** 10.3389/fnagi.2021.747050

**Published:** 2021-10-06

**Authors:** Alessandro Arrigo, Emanuela Aragona, Andrea Saladino, Davide Arrigo, Federico Fantaguzzi, Maurizio Battaglia Parodi, Francesco Bandello

**Affiliations:** ^1^Department of Ophthalmology, Scientific Institute San Raffaele Hospital, Milan, Italy; ^2^School of Medicine, University of Messina, Messina, Italy

**Keywords:** glaucoma, neurodegenerative disease, cognitive impairment, neurodegeneration, neuroinflammation, neuroprotection, OCT, OCTA

## Abstract

**Background**: Glaucoma is a chronic, vision-threatening disease, and a major cause of legal blindness. The current view is no longer limited to the progressive optic nerve injury, since growing evidence strongly support the interpretation of glaucoma as a complex neurodegenerative disease. However, the precise pathogenic mechanisms leading to the onset and progression of central nervous system (CNS) impairment, and the functional consequences of this damage, are still partially understood. The main aim of this review is to provide a complete and updated overview of the current knowledge regarding the CNS involvement in glaucoma, and the possible therapeutic perspectives.

**Methods**: We made a careful survey of the current literature reporting all the relevant findings related to the cognitive dysfunctions occurring in glaucoma, with specific remarks dedicated on the higher-order visual function impairment and the possible employment of neuroprotective agents.

**Results**: The current literature strongly support the interpretation of glaucoma as a multifaceted chronic neurodegenerative disease, widely affecting the CNS. The cognitive impairment may vary in terms of higher-order functions involvement and in the severity of the degeneration. Although several neuroprotective agents are currently available, the development of new molecules represents a major topic of investigation for future clinical trials.

**Conclusions**: Glaucoma earned the right to be fully considered a neurodegenerative disease. Glaucomatous patients may experience a heterogeneous set of visual and cognitive symptoms, progressively deteriorating the quality of life. Neuroprotection is nowadays a necessary therapeutic goal and a future promising way to preserve visual and cognitive functions, thus improving patients’ quality of life.

## Introduction

The term “glaucoma” refers to a group of optic neuropathies characterized by degeneration of retinal ganglion cells (RGCs) and their axons. Their damage lead to the progressive thinning of retinal nerve fibers layer (RNFL) and cupping of the optic nerve head (ONH). Structural changes result in characteristic visual field (VF) alterations, up to complete blindness. Glaucoma is considered a leading cause of blindness worldwide and it is estimated that the number of affected people will increase to approximately 111.82 million in 2040 (Pascolini and Mariotti, 2012; Tham et al., 2014). The main cause of this is that life expectancy is growing, and age is one of the major risk factors for glaucoma (Pascolini and Mariotti, 2012; Tham et al., 2014). The prevalence of primary open-angle glaucoma (POAG), the most common form of glaucoma in the Caucasians, increases significantly with age and many patients develop POAG around 60 years old, reaching a remarkably high incidence (at least 7%) in the Barbadian population (Wensor et al., 1998; Gordon et al., 2002; Leske et al., 2004; European Glaucoma Prevention Study (EGPS) Group, 2007). Ocular hypertension (OHT) is an important risk factor in the development of glaucoma and in its progression (The AGIS Investigators, 2000; Gordon et al., 2002; Heijl et al., 2002). According to the mechanical theory of glaucoma, OHT is the result of a backward displacement of the lamina cribrosa, which in turn would damage the axons of RGCs, compressing them between its meshes. Whereas increased IOP is likely to explain the pathogenesis of optic nerve damage in certain types of glaucoma, this may not be true for some other forms. In particular, normal tension glaucoma (NTG), which afflicts approximately 15–25% of glaucomatous patients, is a clinical condition in which, despite pathologic cupping of the ONH and characteristic VF alterations, IOP is within the normal range (Collaborative Normal-Tension Glaucoma Study Group, 1998; Kim and Park, 2016; Mallick et al., 2016). A relevant proportion of patients is characterized by different patterns of progression; the estimated prevalence of fast-progressors is between 4–10%, and this subtype of patients is characterized by high risk of visual disability (Chauhan et al., 2014; Kirwan et al., 2014; Jammal et al., 2021). To explain NTG-related damages, various non-IOP dependent pathogenetic mechanisms have been proposed, like chronic hypoxia and ischemia, increased intracranial pressure, neuronal glutamate-induced excitotoxicity, mitochondrial dysfunction, oxidative stress, and autoimmunity (Hayreh, 1985; Flammer, 1994; Morgan et al., 1995, 1998, 2002; Romano et al., 1995, 1999; Dreyer et al., 1996; Tezel et al., 1998, 1999; Osborne et al., 2001; Flammer et al., 2002; Gherghel et al., 2005; Grieshaber and Flammer, 2005; Abu-Amero et al., 2006; Grus et al., 2006; Salt and Cordeiro, 2006; Ju et al., 2007; Berdahl et al., 2008a, b; Ren et al., 2010, 2011; Jonas, 2011; Chrysostomou et al., 2013; Siaudvytyte et al., 2014, 2015). Taking together all these factors, a more comprehensive view of glaucoma should be considered, assuming neurodegeneration as a key component of glaucoma pathogenesis. Indeed, many of the above-mentioned mechanisms have been proposed to explain the pathogenesis of neurodegenerative diseases of the central nervous system (CNS). Chronic hypoxia and ischemia account for the development of vascular dementia, which is the second most common cause of irreversible cognitive dysfunction after Alzheimer disease (AD). Excitotoxicity phenomena have been implicated in post-stroke cellular damage and in certain neurodegenerative diseases such as Huntington’s disease, amyotrophic lateral sclerosis (ALS), Parkinson’s disease (PD), and AD (Szatkowski and Attwell, 1994; Rego and Oliveira, 2003). In addition, glaucoma shares some characteristics with neurodegenerative diseases: increased incidence with age, insidious onset, progressive deterioration, and, frequently, a recognized genetic predisposition. This latter is characterized by the involvement of several genes, including myocilin, cytochrome P450 B1 and optineurin (Park et al., 2007; Kumar et al., 2016, 2017). In this complex scenario, this review would explore the clinical similarities between neurodegenerative disorders and glaucoma, the features that seem consistent with neurodegenerative mechanisms characterizing the pathogenesis of glaucoma, and the CNS changes occurring in glaucoma.

## Methods

We searched all English language and human subject articles using keywords search of MEDLINE library. The keywords included the following: glaucoma, open angle glaucoma, normotensive glaucoma, OHT, neurodegeneration, neurodegenerative disease, neuroplasticity, neuroretina, optic nerve, ONH, multimodal imaging, neuroimaging. All the references were carefully examined by two expert researchers (AA,EA) which collected and ordered all the relevant information, considering the main topic of this review as expressed in the manuscript title.

## Multimodal Imaging of The Eye Applied on Glaucoma

**Figure 1:**
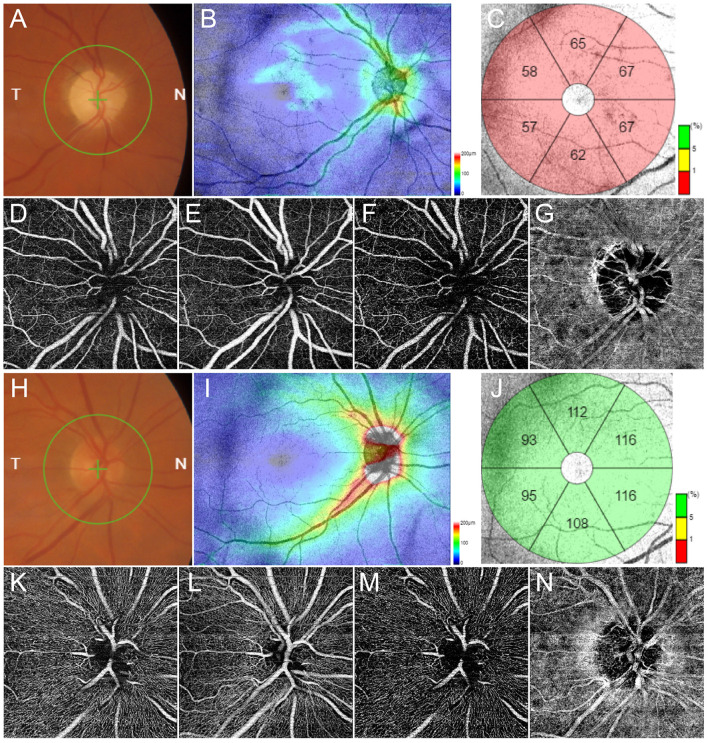
Multimodal imaging in glaucoma. Fundus examination reveals an evident excavation of the optic disc **(A)**. Structural optical coherence tomography (OCT) detects a remarkable thinning of retinal nerve fibers layer (RNFL) **(B)** and ganglion cells layer (GCL) **(C)**. OCT angiography (OCTA) shows rarefied radial peripapillary capillary and deep capillary plexa (**D,F** respectively), and preserved superficial capillary plexus and choriocapillaris (**E,G** respectively). Multimodal imaging findings in a normal control is shown in **(H–N)**, respectively.

Multimodal imaging radically changed the diagnostic approach to posterior segment diseases. This is intended as a set of non-invasive tools assessing different morphological characteristics of the retina and the ONH. Fundus autofluorescence is able to detect the light absorption and emission properties of retinal fluorophores characterizing normal retina and pathological conditions (Schmitz-Valckenberg et al., 2008). Although representing a very important diagnostic tool in retinal diseases, its usage in glaucoma setting is still limited. However, some studies describing ONH autofluorescence changes occurring in glaucoma and the correlation with retinal fibers thinning suggest the utility of this multimodal imaging technique also in glaucoma setting (Viestenz et al., 2006; Reznicek et al., 2013). Optical coherence tomography (OCT) is a laser-based technique able to investigate reflectivity properties of retinal structures and to provide histology-like information (Thomas and Duguid, 2004). It’s role in glaucoma setting is fundamental since it allowed to quantitatively detect ganglion cells layer (GCL) and RNFL alterations and to progressively monitor their evolution with very high accuracy (Leung et al., 2010; Langenegger et al., 2011; Tong et al., 2021). It represents an extremely sensitive, reliable, and reproducible diagnostic approach, and nowadays is of very high utility in the diagnosis and management of glaucoma (Figure 1; Leung et al., 2010; Tong et al., 2021). A recent evolution of OCT is represented by OCT angiography (OCTA), which is able to detect motion signal coming from erythrocytes and to reconstruct intraretinal capillaries (Spaide et al., 2018). If OCTA is of great utility in other retinal diseases, its role in glaucoma setting is still limited. Several previous papers tried to detect early vascular biomarkers of glaucoma onset and progression, demonstrating significant correlations between alterations of the deep capillary plexus and the radial peripapillary capillaries with structural OCT changes and VF damages, thus reinforcing the role of OCTA employment in glaucoma diagnostic workup (Lee et al., 2016; Spaide et al., 2018; Van Melkebeke et al., 2018; Bojikian et al., 2019; Li et al., 2021). A recent report of the American Academy of Ophthalmology highlighted how the quantitative evaluation of vessel density alterations through OCTA may complement the other diagnostic modalities in glaucoma setting (Figure 1; WuDunn et al., 2021).

## Ophthalmic Manifestations of Neurodegenerative Diseases

The eye is often defined as a “window into the brain,” for the big amount of neuroanatomical and vascular information achievable by means of non-invasive diagnostic modalities. Several ocular and visual manifestations have been previously described as complained by patients affected by mild cognitive impairment (MCI) and AD, including extraocular muscles impairment, pupillary defects, RNFL and GCL thinning and visual alterations (for example contrast sensitivity, color vision, VF, visuomotor coordination; Hinton et al., 1986; Trick et al., 1995; Lakshminarayanan et al., 1996; Rizzo et al., 2000; Gilmore et al., 2006; Scinto, 2007; Garbutt et al., 2008; Kavcic et al., 2011; Lee and Pai, 2012; Risacher et al., 2013; Coppola et al., 2015; Liu et al., 2015). In this context, the eye is not a passive viewer of the neurodegenerative cascade occurring in the CNS but is directly involved in MCI/AD pathogenesis. Several studies reported wide accumulations of amyloid-beta precursor protein and β-amyloid within the inner retinal layers (Ning et al., 2008; Koronyo et al., 2012). Furthermore, OCTA was able to detect statistically significant alterations of the intraretinal vascular network in AD patients (van de Kreeke et al., 2020; Wang et al., 2021). The level of evidence regarding the role of retinal diagnostic tools for the early detection of AD is growing, thus promoting the eye as an early biomarker of neurodegenerative disorders (Lim et al., 2016; Cerquera-Jaramillo et al., 2018; Colligris et al., 2018). Similar considerations may be done also considering other neurodegenerative diseases, first of all PD. Although it was mainly considered as a motor disorder, the current point of view regarding PD is changing, nowadays considering this as a complex neurodegenerative disease (Armstrong, 2008). Visual manifestations in PD include a widespread set of alterations involving almost all eye and visual system components (Armstrong, 2011). Interestingly, morpho-functional changes and visual dysfunctions may occur also many years before the onset of motor disorders, both involving the eye and the intracranial visual system, thus strongly suggesting the adoption of ocular findings as early biomarkers of PD onset (Armstrong, 2011; Arrigo et al., 2017a, b, 2018; Guo et al., 2018; Cuerca, 2019; Sung et al., 2019). All these findings provide the basis to explain the wide range of visual alterations complained by patients affected by neurodegenerative diseases. In Table 1 the main visual dysfunction described in two of the most important and representative neurodegenerative diseases, namely AD and PD, are shown. If in this section we described the eye as a structure damaged by pathogenic sources having their primary site in the brain, it should be considered that primary degeneration of ocular structures may induce remarkable changes of the CNS, thus placing the basis for assessing that the neurodegeneration in the eye may somehow have an impact on the neurodegeneration in the brain. The CNS changes occurring as the consequence of primary eye disorders are discussed in the next sections.

**Table 1:** Main visual system alterations in neurodegenerative diseases.

Alzheimer Disease	Parkinson Disease
Visual acuity reduction	Visual acuity reduction
Visual field alterations	Visual field alterations
Color discrimination impairment	Color discrimination impairment
Electrophysiology alterations	Eye movements function deficits
Contrast and temporal discrimination impairment	Pupil function and reactivity decreases
Visual and visuo-motor tasks alterations	Electrophysiology alterations
Visual hallucinations	Contrast and temporal discrimination impairment
Posterior pole morpho-functional alterations	Visual and visuo-motor tasks alterations
Balint’s syndrome (simultanagnosia + ocular motor apraxia + optic ataxia)	Visual hallucinations
Corneal nerves alterations	Corneal nerves alterations
Retinal and optic nerve morpho-functional alterations	Retinal and optic nerve morpho-functional alterations
Intracranial visual system morpho-functional changes	Intracranial visual system morpho-functional changes

## Neurodegenerative Manifestations in Ophthalmic Diseases

The other side of the coin is the involvement of the CNS in ophthalmic diseases, representing a topic of growing interest for the clinical and scientific communities (Prins et al., 2016). Indeed, posterior segment disorders can induce neurodegenerative and neuroplasticity phenomena within the brain, towards anterograde and retrograde transsynaptic mechanisms. Age-related macular degeneration is associated with deep degenerative changes of the intracranial visual pathways, functional impairment of visual and visuomotor tasks and CNS connectivity network modifications induced by neuroplasticity phenomena (Lešták et al., 2013; Rosengarth et al., 2013; Hernowo et al., 2014). Similar changes may occur also in inherited retinal dystrophies, where white matter network changes and cortical remapping have been described (Olivo et al., 2015; Ferreira et al., 2016; Rita Machado et al., 2017). If visual system changes occur in the first stages to try to compensate the progressive visual degeneration, the later stages of retinal diseases are characterized by extensive morpho-functional impairment also of the intracranial visual system (Nuzzi et al., 2020). Although still poorly investigated, the profound interconnection between the eye and CNS may have extremely important implications for a deeper understanding of visual processing mechanisms and for ocular and CNS diseases.

## Glaucoma as A Neurodegenerative Disease

The eye and the brain are strictly interconnected through the optic nerve and even growing evidence support the existence of a strong morpho-functional interconnection between these two compartments, allowing to share common pathogenic neurodegenerative pathways and manifestations. The most used models to assess the similarities between eye and brain neurodegeneration are glaucoma and AD. First of all, these two diseases are characterized by the similar retinal sites of damage, namely GCL and RNFL (Blanks et al., 1996a, b). Ganglion cells loss is mainly caused by increased oxidative and metabolic distress, neuroinflammation-mediated damage, and increased glial cells reactivity (McKinnon, 2012; Sivak, 2013; Jindal, 2015; Wei et al., 2019; Volkert and Crowley, 2020).

In this intricated scenario, glaucoma can be fully considered as a complex neurodegenerative disease. Indeed, the pathogenic features characterizing the progressive morpho-functional disruption of the neuroretinal structures and the patterns of involvement of the CNS in glaucoma share many common characteristics with other neurodegenerative disorders. A key molecular mechanism occurring in glaucoma is the neuroinflammation, a complex cascade of multifactorial events leading to the damage of neuroretinal structures (McKinnon, 2012; Wei et al., 2019; Jiang et al., 2020; Quaranta et al., 2021). Likely AD, glaucomatous damage is promoted by the activation of tumor necrosis factor-alpha and complement system pathways (Stasi et al., 2006; Tezel, 2008). Several autoantibodies of neuroinflammation and neurodegeneration have been found highly present in glaucomatous patients, with different patterns of expression between POAG and NTG (Vu et al., 2019). In addition, other circulating biomarkers associated with neurotrophy, neuroprotection and oxidative stress have been found altered in glaucoma, including serum homocysteine, vitamin B12, folic acid and endothelin-1 (ET-1; Cumurcu et al., 2006; Turgut et al., 2010; Türkcü et al., 2013; López-Riquelme et al., 2015). Another remarkable similarity between glaucoma and AD is represented by the presence of amyloid precursor protein accumulations in glaucomatous eyes (McKinnon et al., 2002).

Amyloid-β protein and its precursor are a major pathogenic source of damage, turning out to be at the center of several pathogenic cascades leading to the activation and promotion of neurodegeneration. The role of amyloid-β in the retina is still unknown, although previous authors hypothesized an antimicrobial activity, similarly to what found within the CNS (Wostyn et al., 2015; Kumar et al., 2016; Naaman et al., 2020). On the other side, amyloid precursor protein is known to exert several neuroprotective activities, including the support to synaptogenesis and the promotion of neuronal development and survival (Jessen et al., 2015). This is true when amyloid-β and amyloid precursor protein maintain monomeric configuration. However, it has been described the spontaneous aggregation of amyloid-β into dimers, trimers, and oligomers; the insoluble form of amyloid-β oligomers lead to the formation of protofibrils, fibrils, and insoluble amyloid plaques which are toxic for retinal neurons, activating several pathologic cascades similarly to what happen within the brain in AD (Naaman et al., 2020). Furthermore, amyloid precursor protein physiologically interacts with other molecules, including integrins and receptor tyrosine kinase; the impairment of this metabolic network was associated with tau-related excitotoxicity causing synaptic and axonal failure.

In glaucoma, a possible pathogenic hypothesis of amyloid-β accumulation has been based on the dysfunction of the retinal glymphatic system (Wostyn et al., 2015). Indeed, the retina as well as the brain have no lymphatic vessels; for this reason, glial cells may work as scavenger elements, towards the aquaporins system, to allow the drainage of fluids and molecules (Jessen et al., 2015). The glymphatic system involvement in glaucoma might be based on the impaired outflow and stasis caused by changes of the pressure barrier across the lamina cribrosa. This latter is the result of a delicate balance of the intraocular and the intracranial pressures; an increased IOP or a decreased intracranial pressure may be the cause of the glymphatic system disruption (Wostyn et al., 2016; Wang et al., 2020; Wang and Mao, 2021).

**Figure 2:**
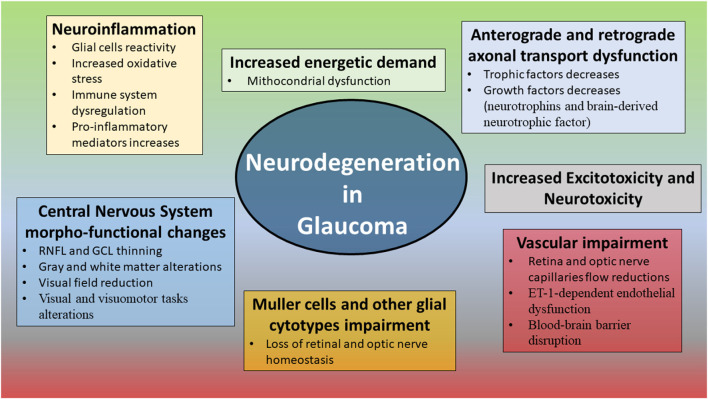
Main alterations associated with neurodegenerative mechanisms in glaucoma.

Several evidences described other interesting molecular mechanisms significantly associated with the onset and progression of glaucomatous damage, including the disbalance of neurotrophic factors production, the reactive activation of glial cells, increased oxidative stress, dysregulation of the immune system, and energetic demand caused by mitochondrial dysfunction (Tezel and Wax, 2000; Tezel, 2006, 2021; Ju et al., 2009; Baudouin et al., 2021). In this context, increasing IOP values may act as a trigger for the onset of these pathogenic mechanisms, although these may independently occur, like in NTG (Munemasa and Kitaoka, 2013). Even growing studies are defining a multifactorial pathogenic scenario leading to the onset and progression of NTG, where several mechanisms including vascular dysregulation, mainly caused by ET-1-dependent endothelial dysfunction (Moore et al., 2008), glial cells reactivity, leading to neuroinflammatory and oxidative distresses (Tezel and Wax, 2004; Mozaffarieh and Flammer, 2013), impairment of exchanges towards the lamina cribrosa (Tezel and Wax, 2004; Rao et al., 2007, 2008), and blood-brain barrier disruption (Hofman et al., 2001; Grieshaber and Flammer, 2007). Other demonstrated neurotrophic deprivations regarded the reduced production and release of neurotrophins and brain-derived neurotrophic factor (Vrabec and Levin, 2007). These molecules are mainly released by Muller cells and are fundamental for neuronal cell survival (Vrabec and Levin, 2007). The involvement of Muller cells and other glial cytotypes resulted extremely important in the pathogenesis of glaucoma, leading to the progressive loss of retinal and optic nerve homeostasis, promoting neuroinflammation and neurodegeneration processes (Seki et al., 2005; Vrabec and Levin, 2007; Seitz et al., 2013; Chong and Martin, 2015). It is worth of notice that all these mechanisms are far from the pathogenic cascade which can be induced by the mere IOP value increases. All the main mechanisms characterizing neurodegeneration in glaucoma are shown in Figure 2. In addition to all these factors making the neurodegenerative pathogenesis of glaucoma extremely complex, growing findings add a further cause of damage, represented by the impairment of the homeostatic brain and eye fluid balance. Indeed, decreased ocular and brain blood flows, cerebrospinal fluid disbalances, higher vascular resistance and diffuse cerebral hypoperfusion/ischemic changes have been described in glaucoma patients (Harris et al., 2003; Heringa et al., 2013). This chronic sufferance is not limited to the retina and the ONH.

Indeed, what is of great interest is the number of changes and damages occurring at the level of the CNS. Magnetic resonance imaging (MRI) techniques described widespread modifications of the white matter tissue and the brain connectivity in patients affected by glaucoma, with strong correlations with RNFL/GCL impairment and VF alterations (Garaci et al., 2009; El-Rafei et al., 2013; Omodaka et al., 2014; Zhao et al., 2021). Similarly, several gray matter thinning of the visual cortex have been also described (Zhang et al., 2015; Zhou et al., 2017; Giorgio et al., 2018). Gray matter thinning involved also other brain regions over than the occipital cortex, including lingual gyrus, calcarine gyrus, postcentral gyrus, superior frontal gyrus, inferior frontal gyrus, rolandic operculum, cerebellar cortex, and hippocampus (Li et al., 2012, 2020; Frezzotti et al., 2014). On the other side, some other gray matter regions associated with visual processing turned out to be thicker in patients with glaucoma, including middle temporal gyrus, inferior parietal gyrus, angular gyrus, midbrain, brainstem, frontal gyrus, cerebellar vermis and thalamus (Jiang et al., 2017; Giorgio et al., 2018; Kasi et al., 2019). The wide involvement of extra-occipital regions may justify previous findings reporting significant alterations of white matter connections involved in visual and visuomotor tasks, and in high order functions, including the inferior fronto-occipital fasciculus, the longitudinal and the inferior frontal fasciculi, putamen, caudate nucleus, anterior and posterior thalamic radiations and anterior and posterior limbs of the internal capsule (Zikou et al., 2012; Williams et al., 2013). Although the whole CNS alterations significantly correlated with the stage of glaucoma and disease’s severity (Wang et al., 2021), many studies highlighted how gray and white matter extensive changes may be detected already at early stages of glaucomatous disease (Yu et al., 2013; Frezzotti et al., 2016; Kasi et al., 2019) thus supporting the hypothesis that the CNS involvement is not only a secondary phenomenon related with optic nerve damage, but it may represent the result of an active cascade of pathological mechanisms evolving independently from the optic nerve degeneration. Taking together all the above described visual and non-visual brain connectivity alterations, it may be assumed that glaucomatous patients may suffer from brain processing dysfunctions localized at different levels, including visual and visuomotor tasks, memory and emotion, working memory and attention, default mode network and other multimodal brain functions (Zhang et al., 2016; Wang et al., 2017; Nuzzi et al., 2018; Giorgio et al., 2020).

## Cognitive Impairment in Glaucoma

Cognitive impairment is a relatively novel concept described in glaucoma, which is gaining increased attention and importance. It represents a progressive decline of memory and multimodal high order brain functions, which is usually categorized, accordingly with the gravity of the cognitive deterioration, as MCI or dementia (Petersen, 2011; Jongsiriyanyong and Limpawattana, 2018). An increasing body of literature reported high frequency of cognitive impairment in patients affected by glaucoma than in normal controls (Harrabi et al., 2015; Su et al., 2016; Maurano et al., 2018; Varin et al., 2020). On the other side, patients with AD and dementia had an increased risk of glaucoma onset (Xu et al., 2019; Vidal et al., 2020). The link between these two apparently different conditions is further reinforced by the common finding of RNFL/GCL thinning and visual gray and white matters morpho-functional impairments (Iseri et al., 2006; Tamura et al., 2006; Ascaso et al., 2014; Jones-Odeh and Hammond, 2015). The relationship between retinal and CNS alterations is strong and support the role of OCT-based technology for the early diagnosis of cognitive dysfunctions and neurodegenerative diseases (Biscetti et al., 2021). A well-known glaucoma-related finding is the thinning of the lamina cribrosa, a complex tissue surrounding the ONH and providing structural and functional supports to the ganglion cells. Interestingly, lamina cribrosa thinning was found significantly associated with worse global cognitive function [measured by Mini-Mental State Exam (MMSE) score], independently from the severity of glaucoma (Lee et al., 2020). The cognitive impairment in glaucoma, measured by means of different cognitive assessment tests, opens new management issues with respect to the reliability of VF examination; many studies showed a significant relationship between neurocognitive decline and VF variability, placing a solid basis for a remarkable structure-function relationship mismatch which may interfere with the proper management of glaucomatous patients (Diniz-Filho et al., 2017; Honjo et al., 2017; Raman et al., 2019). Indeed, decreased VF test reliability can delay the detection of true progression of the disease, potentially resulting in irreversible visual function loss. Conversely, false VF worsening, when no true changes occur, may lead to unnecessary treatment changes with potential negative effects for patients. On the other hand, visual function decrease may contribute to cognitive deterioration, because of leading patients to lower daily activity levels and progressive loss of self-sufficiency, especially in older ages (Bassuk et al., 1999; Wilson et al., 2002). As above described, the involvement of the CNS and the impairment of cognitive functions in glaucoma is ruled by extensive changes of functional connectivity networks, with increased phenomena of neuroplasticity and neurodegeneration. Because of its relatively recent discovery, few studies have been conducted focusing on cognitive dysfunctions in glaucoma. However, if considering the importance of the visual function in cognitive tasks, this research field should be object of further investigations in glaucoma. Just to provide an example, it was demonstrated that sensory functions impairments have a direct role in cognitive aging, since good sensory functions, with particular regard to visual task, were found strongly predictive of the cognitive performance (Glass, 2007). Interestingly, each visual function has a different impact on cognitive impairment, with contrast sensitivity seeming to have the strongest relationship with cognitive tests scores (Varadaraj et al., 2021). It is worth of notice that glaucomatous patients are characterized by significantly higher impairment of contrast sensitivity, with respect to normal aging (McKendrick et al., 2007). On this basis, it is assumable that glaucoma could alter cognitive performance, especially in advanced stages (McCoskey et al., 2018; Mullany et al., 2021). This is the reason why future research should be focused on the deep assessment of the pathologic cascades occurring beyond the eye, in order to draw more definite conclusion about the impact of glaucomatous changes on brain high order functions.

Based on what described in this review, the careful assessment of CNS involvement might pave the stone for future diagnostic and therapeutic challenges, guiding clinicians and researchers on a more comprehensive evaluation of the pathophysiology of glaucoma and improvements in the management of visual and non-visual alterations complained by glaucomatous patients.

## Future Perspectives

The current management of glaucoma is mainly based on IOP-lowering medications and IOP-lowering surgical approaches. Although IOP control is a crucial step for the management of glaucomatous patients, what discussed in the present review opens new perspectives in the fields of diagnostic workup and treatment of glaucoma. The awareness regarding the extensive involvement of the CNS, causing the onset of several non-ocular dysfunctions, offers the basis for the development of new treatment strategies dedicated on neuroprotection. Since glutamate-induced excitotoxicity is a main factor associated with glaucomatous neurodegeneration, a possible therapeutic approach might be based on the employment of glutamate inhibitors, such as dizocilpine maleate, memantine and Bis(7)-tacrine (Lipton, 2003; Guo et al., 2006; Fang et al., 2010). These N-methyl D-aspartate (NMDA) receptor blockers act as potent inhibitors of glutamate, although the level of evidence regarding their positive effect in glaucoma setting is not still high and sometimes these molecules resulted toxic for neurons. Furthermore, the administration of memantine was not associated with statistically significant lowering of glaucoma progression. Another class of molecules currently under investigation is represented by ginkgo biloba extracts. Ginkgo biloba already showed positive effects against cognitive impairment in neurodegenerative diseases, such as AD, and positive findings have been reported in glaucoma, thus suggesting a therapeutic role for glaucomatous patients (Quaranta et al., 2003). Brimonidine is a selective alpha-2 receptor adrenergic agonist binding receptors localized within the retina and increasing retinal metabolism and neuronal growth. The rationale regarding the use of brimonidine in glaucoma is based on the neuroprotective role that this molecule should have for retinal ganglion cells, thus preventing their degeneration (Kalapesi et al., 2005). Other therapeutic perspectives are based on the employment of antioxidants, vasoprotective agents, other anti-neurotoxic molecules, such as nitric oxide synthase inhibitors and calcium-channel blockers, other neurotrophic factors, such as BDNF and ciliary neurotrophic factor, and stem cells approaches (Doozandeh and Yazdani, 2016). The main clinical trials dedicated on neuroprotective therapeutic approaches in glaucoma setting are listed in Table 2. It is worth of notice that two interventional studies are currently active, focused on NT-501 encapsulated cell therapy implant for increasing the production of ciliary neurotrophic factor (NCT02862938) and on testing GlaucoCetin nutraceutical approach (NCT04784234), respectively. With respect to clinical trials focused on cognitive impairment in glaucoma, few studies are currently registered on https://clinicaltrials.gov/. NCT01303939 is a MRI-based clinical trial started in 2011 and ended in 2013 adopting neuroimaging approaches to assess CNS involvement in glaucoma. NCT03333096 is a trial started in 2017, focused on drive fitness and cognitive performance including attention assessments in patients affected by glaucoma and MCI by means of specific neuropsychological tests. NCT03318549 is a clinical trial started in 2017 with the aim of administering visual tasks performance tests to assess visual functional impairment in different eye disorders, including glaucoma.

**Table 2:** Clinical trials dedicated on neuroprotection in glaucoma (from https://clinicaltrials.gov/).

Clinical trials dedicated on neuroprotection in glaucoma					
Number	Study Title	Identifier	Drug/Approach	Target	Status
1	A Randomized, Sham Controlled, Masked Phase II Study to Evaluate the Safety and Efficacy of Intravitreal Implantation of NT-501 Encapsulated Cell Therapy for the Treatment of Glaucoma	NCT02862938	NT-501 encapsulated cell therapy implant	Production of ciliary neurotrophic factor	Active, not recruiting
2	A Prospective Randomized Controlled Trial of GlaucoCetin vs. Placebo in Glaucoma Patients With Visual Field Loss.	NCT04784234	GlaucoCetin	Neuroprotection	Recruiting
3	A Multicenter, Double-Masked, 2-Arm Parallel Group Study Comparing the Effect of Brimonidine 0.2% vs. Timolol 0.5% on Visual Field Stability in Patients With Low-Pressure Glaucoma	NCT00317577	Brimonidine	Neuroprotection	Completed 2004
4	Topical Brimonidine vs. Argon Laser Trabeculoplasty in Progressing Human Glaucoma. A Prospective Randomized Clinical Trial.	NCT00466479	Brimonidine	Neuroprotection	Completed 2007
5	A Phase II Study to Investigate the Safety, Efficacy, and Pharmacokinetic Profile of Twice-Daily DNB-001 in Previously Untreated Patients With Elevated Intraocular Hypertension	NCT00683501	DNB-001	Ion channel modulator	Completed 2008
6	Impact of Oral Versatile Antioxidants on Glaucoma Progression:Comparative Early Results	NCT01544192	Gingko Biloba; α-tocopherol	Neuroprotection	Completed 2012
7	Impact of Oral Versatile Antioxidants on Glaucoma Progression:Comparative Early Results	NCT01544192	Gingko Biloba; α-tocopherol	Neuroprotection	Completed 2012
8	CNTF Cell Implants For Glaucoma: A Phase I Study	NCT01408472	NT-501 encapsulated cell therapy implant	Production of ciliary neurotrophic factor	Completed 2014

## Conclusion

This review tried to collect the main findings supporting the existence of many elements in common between neurocognitive dysfunctions in glaucoma and neurodegenerative diseases, leading to MCI/dementia. The eye and the CNS are closely interconnected, placing the basis for the onset of visual symptoms in neurodegenerative diseases and, conversely, of neurocognitive dysfunctions in ophthalmic disorders. Glaucomatous patients are characterized by a wide range of still poorly investigated cognitive dysfunctions. These alterations, governed by neuroinflammatory and neurodegenerative mechanisms, may have a strong impact on patients’ quality of life and may remarkably interfere with the proper diagnostic and therapeutic management of glaucoma. Although still representing a novel research field, the vision of glaucoma as a complex neurodegenerative disease would open new diagnostic and therapeutic routes, more focused on the involvement of the neuroretinal and the CNS structures. Further studies should be focused on a deeper assessment of CNS alterations in glaucoma and on the development of new neuroprotective therapeutic strategies.

## Author Contributions

AA and EA: review design, data analysis, data interpretation, and manuscript draft. AS, DA, and FF: data acquisition and data analysis. MB and FB: data interpretation, manuscript revision, and study supervision. All authors contributed to the article and approved the submitted version.

## Conflict of Interest

FB consultant for Alcon (Fort Worth, Texas, USA), Alimera Sciences (Alpharetta, Georgia, USA), Allergan Inc. (Irvine, California, USA), Farmila-Thea (Clermont-Ferrand, France), Bayer Shering-Pharma (Berlin, Germany), Bausch and Lomb (Rochester, New York, USA), Genentech (San Francisco, California, USA), Hoffmann-La-Roche (Basel, Switzerland), Novagali Pharma (Évry, France), Novartis (Basel, Switzerland), Sanofi-Aventis (Paris, France), Thrombogenics (Heverlee, Belgium), Zeiss (Dublin, California, USA). The remaining authors declare that the research was conducted in the absence of any commercial or financial relationships that could be construed as a potential conflict of interest.

## Publisher’s Note

All claims expressed in this article are solely those of the authors and do not necessarily represent those of their affiliated organizations, or those of the publisher, the editors and the reviewers. Any product that may be evaluated in this article, or claim that may be made by its manufacturer, is not guaranteed or endorsed by the publisher.
